# Genetic and Drug Inhibition of LDH-A: Effects on Murine Gliomas

**DOI:** 10.3390/cancers14092306

**Published:** 2022-05-06

**Authors:** Masatomo Maeda, Myat Ko, Mayuresh M. Mane, Ivan J. Cohen, Masahiro Shindo, Kiranmayi Vemuri, Inna Serganova, Ronald Blasberg

**Affiliations:** 1 Department of Neurology, Memorial Sloan Kettering Cancer Center, New York, NY 10065, USA; masatomomaeda@gmail.com (M.M.); mko@wyckoffhospital.org (M.K.); manem@mskcc.org (M.M.M.); shindoum@yukioka.or.jp (M.S.); k.vemuri@rutgers.edu (K.V.); serganoi@mskcc.org (I.S.); 2 Molecular Pharmacology and Chemistry Program, Memorial Sloan Kettering Cancer Center, New York, NY 10065, USA; ivan.cohen@pennmedicine.upenn.edu; 3 Department of Neurosurgery, Nozaki Tokushukai Hospital, Osaka 5740074, Japan; 4 Human Oncology and Pathogenesis Program, Memorial Sloan Kettering Cancer Center, New York, NY 10065, USA; 5 Department of Radiology, Memorial Sloan Kettering Cancer Center, New York, NY 10065, USA; 6 Gerstner Sloan Kettering Graduate School of Biomedical Sciences, Memorial Sloan Kettering Cancer Center, New York, NY 10065, USA; 7 Center for Cellular Immunotherapies, Perelman School of Medicine, University of Pennsylvania, Philadelphia, PA 19104, USA; 8 Department of Neurosurgery, Yukioka Hospital, Osaka 5740074, Japan; 9 Department of Genetics, Rutgers University, New Brunswick, NJ 08901, USA; 10 Weill Cornell Medicine, New York, NY 10021, USA

**Keywords:** glioblastoma, LDH-A shRNA knockdown, GNE-R-140, LDH isoenzymes, LDH-A and LDH-B immunohistochemistry, tumor growth, lactate, immunocompetent host animals, RNA seqence analyses

## Abstract

**Simple Summary:**

Three different murine glioma cell lines were modified to downregulate expression of the murine *LDH-A* gene using shRNA knockdown (KD) and compared to pharmacologic (GNE-R-140) inhibition of the LDH enzyme complex, and to shRNA scrambled control (NC) cell lines. The effects of shRNA LDH-A knockdown and LDH drug-targeted inhibition (GNE-R-140) on tumor-cell metabolism, tumor growth, and animal survival were similar in each of the cell lines. However, an unexpected increase in the aggressiveness was observed in *LDH-A* KD and GNE-R-140 treated GL261 intracranial gliomas, but not in CT2A and ALTS1C1 i.c. gliomas. Our results show that LDH-A KD and GNE-R-140 treated GL261 cells are better able to metabolize lactate as a primary carbon source through the TCA cycle, and are a net consumer of lactate. These results suggest that inhibition of LDH-A/glycolysis may not be a general strategy to inhibit the i.c. growth of all gliomas, and that metabolic-inhibition treatment strategies need to be carefully assessed, since the inhibition of glycolysis may lead to the unexpected development and activation of alternative metabolic pathways resulting in enhanced tumor-cell survival in a nutrient-limited environment, leading to increased tumor aggressiveness.

**Abstract:**

The effects of the LDH-A depletion via shRNA knockdown on three murine glioma cell lines and corresponding intracranial (i.c.) tumors were studied and compared to pharmacologic (GNE-R-140) inhibition of the LDH enzyme complex, and to shRNA scrambled control (NC) cell lines. The effects of genetic-shRNA LDH-A knockdown and LDH drug-targeted inhibition (GNE-R-140) on tumor-cell metabolism, tumor growth, and animal survival were similar. LDH-A KD and GNE-R-140 unexpectedly increased the aggressiveness of GL261 intracranial gliomas, but not CT2A and ALTS1C1 i.c. gliomas. Furthermore, the bioenergetic profiles (ECAR and OCR) of GL261 NC and LDH-A KD cells under different nutrient limitations showed that (a) exogenous pyruvate is not a major carbon source for metabolism through the TCA cycle of native GL261 cells; and (b) the unique upregulation of LDH-B that occurs in GL261 LDH-A KD cells results in these cells being better able to: (i) metabolize lactate as a primary carbon source through the TCA cycle, (ii) be a net consumer of lactate, and (iii) showed a significant increase in the proliferation rate following the addition of 10 mM lactate to the glucose-free media (only seen in GL261 KD cells). Our study suggests that inhibition of LDH-A/glycolysis may not be a general strategy to inhibit the i.c. growth of all gliomas, since the level of LDH-A expression and its interplay with LDH-B can lead to complex metabolic interactions between tumor cells and their environment. Metabolic-inhibition treatment strategies need to be carefully assessed, since the inhibition of glycolysis (e.g., inhibition of LDH-A) may lead to the unexpected development and activation of alternative metabolic pathways (e.g., upregulation of lipid metabolism and fatty-acid oxidation pathways), resulting in enhanced tumor-cell survival in a nutrient-limited environment and leading to increased tumor aggressiveness.

## 1. Introduction

Glioma or glioblastoma multiforme (GBM) is the most common malignant primary brain tumor, with a median survival of just over 12 months and with limited effective therapy [1]. The astrocytoma is the most aggressive form of GBM, as well as the most common. The malignant transformation of gliomas is associated with alterations in the metabolism of glucose, amino acid, and fatty acid [2]. Understanding the metabolic variations among glioma and normal brain tissue or between different grades of glioma might provide an insight into their malignant behavior and could offer other opportunities toward therapeutic targets. Recently targeting LDH/lactate axis became a promising approach for cancer therapy [3,4,5]. Lactate is produced as a result of lactate dehydrogenase (LDH) activity and was considered as a waste product of glycolysis. However, lactate plays important roles in brain energetics [6]. LDH is a tetrameric enzyme composed of two protein subunits, forming the protein complex approximately 135 kDa [7]. The tetramer can assemble as five separate isozymes by forming all combinations of the M (muscle) form (product of the *LDH-A* gene) or the H (heart) form (product of the *LDH-B* gene) producing: M_4_ (A_4_ = LDH_5_), M_3_H_1_ (A_3_B_1_ = LDH_4_), M_2_H_2_ (A_2_B_2_ = LDH_3_), M_1_H_3_ (A_1_B_3_ = LDH_2_), and H_4_ (B_4_ = LDH_1_) [8]. The expression pattern of LDH-A in GBM analyses have shown the differential mRNA expression of LDH-A between GBM tissues and normal brain tissues. The expression of LDH-A was significantly increased in GBM tissues compared with corresponding normal brain tissues, based on analyses using different databases, and indicated a deregulated expression of LDH-A in GBM [9]. The role and impact of LDH-A expression in different cancer types has been well-explored, whereas the effect of LDH-B expression and its association with LDH-A expression is less well-understood. It is known that the LDH-B promoter is silenced in prostate, gastric, and colon cancers through the hypermethylation mechanism [10,11] and required for the growth of KRAS-dependent lung adenocarcinomas [12].

LDH catalyzes the coordinated interconversion of pyruvate and lactate as well as NADH and NAD+. The properties of LDH isozymes regarding substrate affinity were investigated in in vitro experiments. M-dominated isozymes (*LDH-A*, LDH-5) have 3.5–7 times higher K_m_-values for pyruvate and lactate than the H-dominated forms (*LDH-B*, LDH-1). LDH-1 is inhibited by pyruvate at concentrations above ~0.2 mM, while LDH-5 is minimally affected by pyruvate at concentrations as high as 5 mM [13]. The LDH-1 isozyme is inhibited by lactate above 20–40 mM, while the LDH-5 isozyme is less inhibited by high lactate levels, pointing to functional differences between LDH-1 and LDH-5 isozymes in cellular metabolism. In the brain, astrocytes have high glycolytic metabolism and a greater proportion of the M-type LDH isozyme (LDH-5), whereas neurons have a high oxidative metabolism and a greater proportion of the H-type LDH isozyme (LDH-1) [14].

Therefore, we decided to investigate and compare the impact of LDH-A depletion (both LDH-A shRNA knockdown (KD) and treatment with a specific LDH-A/B inhibitor (GNE-R-140)) on the expression of LDH-B in different murine glioma cell lines and corresponding i.c. tumors. We show that control intracranial GL261 tumors shift from an LDH-A (LDH4 dominant) to an LDH-B (LDH1 dominant) pattern and phenotype following LDH-A KD and treatment with GNE-R-140. These changes in the LDH isoenzyme profile and changes in tumor phenotype were not observed in CT2A or ALTS1C1 NC and LDH-A KD tumors. The level of LDH-A expression and its interplay with LDH-B can lead to metabolic changes and complex interactions between tumor cells and their environment. These interactions need to be carefully assessed, since the inhibition of glycolysis in tumor cells may lead to the activation of other metabolic pathways (e.g., lipid, fatty acid, oxidative) and to phenotypic changes, including increased tumor aggressiveness.

## 2. Materials and Methods

### 2.1. Cells and Culture Conditions

The GL261 murine glioblastoma cell line was originally obtained from NCI depository. The ALTS1C1 murine glioblastoma cell line was kindly provided by Dr. Chiang (Department of Biomedical Engineering and Environmental Sciences, National Tsing Hua University, Taiwan) and the CT2A murine glioblastoma cell line was kindly provided by Dr. Seyfried (Biology Department, Boston College, Boston, MA, USA). These cell lines were cultured in DMEM media supplemented with 25 mM of glucose, 10% FCS, 4 mM glutamine, and penicillin/streptomycin. LDH-A KD (knock-down) and NC (control) cells, derived from each murine glioblastoma cell, were grown in the media described above containing 2.5 mg/L of puromycin.

### 2.2. Generation of LDH-A Knockdown and Control Cell Lines

GL261, CT2A, and ALTS1C1 cells were transfected with SureSilencing shRNA plasmids (QIAGEN, Frederick, MD, USA) to specifically knock down expression of the mouse *LDH-A* gene as we described previously [15,16,17,18,19]. Stably transduced clones (KD cell lines) were developed, along with a control (NC) cell line bearing a scrambled shRNA. Based on our previous experience, we decided to use the most effective shRNA (shRNA-2) from the set of 4 shRNAs to develop LDH-A KD in murine glioma cells. Our previous experience in other cell lines determined that shRNA-2 resulted in the best *LDH-A* knockdown function in murine cells. Although shRNA-3 *LDH-A* knockdown was less effective, the phenotypic changes in cells and tumors were comparable to that obtained with shRNA-2 [15,18]. The transfection of GL261 cancer cells with shRNA-2 resulted in a significant knockdown effect for LDH-A (approximately 30% of that in wild type cells), while bulk CT2A and ALTS1C1 cells transfected with shRNA-2 had a less profound LDH-A knockdown (40–60%) detected by mRNA, proteins levels. To enrich the level of LDH-A knockdown we used a subcloning strategy for CT2A and ALTS1C1 cell lines, while LDH-A GL261 knockdown cells were used as a bulk [19].

### 2.3. Western Blotting

Western blotting for protein expression was performed as described previously [15,16]. RIPA Buffer (25 mM Tris-HCl (pH 7.6), 150 mM NaCl, 1% NP-40, 1% sodium deoxycholate, 0.1% SDS (Thermo Scientific, Waltham, MA, USA) and protease and phosphatase inhibitor cocktail (1:100, Thermo Scientific Halt Protease and Phosphatase Inhibitor Single-Use Cocktail) was used to lyse cell pellets. Bicinchoninic acid assay (BCA Protein Assay Kit, Thermo Scientific) was performed to assess protein concentrations. 10–30 µg of proteins were separated by electrophoresis using a NuPAGE 4–12% Bis-Tris Gradient Gel or NuPAGE 10% Bis-Tris Gradient Gel (Invitrogen, Waltham, MA, USA) and transferred to an Immuno-Blot PVDF Membrane (BioRad, Hercules, CA, USA). Membranes were blocked in 5% non-fat dry milk in Tris-buffered saline—Tween20 buffer and immunoblotted with anti-LDH-A antibody (Cell Signaling, #2012S) at a 1:1000 dilution; LDH-B (Proteintech #14824-1-AP) at a dilution 1:2000 and antiactin antibody (Sigma life science, #A2103) at a 1:5000 dilution. Bound primary antibodies were visualized with Eu-labeled antibody using ScanLater Western Blot Assay kit and SpectraMax ID5 (Molecular Devices, San Jose, CA, USA).

### 2.4. LDH Enzyme Activity and Lactate-Glo Assay

Total LDH enzyme activity was assessed using the Cytotoxicity Detection Kit PLUS (LDH) (Roche Diagnostics). 10,000 cells were plated in 96-well plates and incubated (37 °C, 5% CO_2_, humidified incubator) for 1 h. LDH enzyme activity from lysed cells was measured using absorbance assessed at 490 nm using Spectramax ID5 (Molecular Devices, USA).

For other biochemical assays, 20,000 or 200,000 cells were seeded in 96-well or 6-well plates correspondingly and incubated (37 °C, 5% CO_2_, humidified incubator) for 2–4 h for their attachment. After the attachment, the media was changed to DMEM media containing 0 or 5 mM glucose, 0 or 2 mM pyruvate, and 0 or 10 mM Na-lactate. After the 24 h incubation, media was aspirated, and cells were washed with PBS and collected by centrifugation. Lactate was extracted by using 50 mM Tris (pH-7.4) solution with following 0.6 N HCl. In order to measure the intracellular lactate level from cells we used the Lactate-Glo kit (Promega, Madison, WI, USA).

### 2.5. LDH Zymography

LDH zymography was used to detect tissue-specific differences in LDH isoenzymes. We were able to observe 5 isozyme bands in the active state as previously described [20]. Based on the different electrophoretic motilities of the isoenzymes, they can be identified as LDH1 (B4 or H4), LDH2 (B3A1 or H3M1), LDH3 (B2A2 or H2M2), LDH4 (B1A3 or H1M3), and LDH5 (A4 or M4). We used a buffer at pH 8.6 for the best separation of the five LDH isoenzymes [13,14].

### 2.6. Metabolic Extracellular Flux Analysis

Glycolytic and mitochondrial activity of cells was measured using a Seahorse XF96 Extracellular Flux Analyzer (Agilent Seahorse XF Technology, Billerica, MA, USA). Cells were seeded at 25,000–30,000 cells per well with standard growth media using Seahorse XF96 96-well plates; the cells were allowed to attach over 6 h at 37 °C in an incubator (95% air/5% CO_2_). Total proton efflux rate (PER) was measured by plotting proton efflux as a function of time (pmol/min). Oxygen consumption rate (OCR) was measured as the change in oxygen content of the media as a function of time (pmol/min). Data was normalized to the number of cells in each well. Data from 3 independent experiments were analyzed using Seahorse Wave Desktop Software and compiled together using GraphPad Prism 7.

### 2.7. mRNA Gene Expression Profile Analysis

LDH-A knockdown was verified by two approaches. First, a quantitative digital droplet PCR (ddPCR) was performed for LDH-A and LDH-B by the Genomics Core Laboratory at MSKCC. For RNA purification, cells were grown for 48 h (exponential growth phase). RNA was isolated using the RNeasy total RNA isolation kit (QIAGEN, catalog No. 74104), following the manufacturer’s protocol. Second, RNA sequencing was performed after RNA extraction, library preparations, and RNA-sequencing reactions conducted at GENEWIZ, LLC. (South Plainfield, NJ, USA). Total RNA was extracted from frozen cell-pellet samples using Qiagen RNeasy Plus Universal mini kit following manufacturer’s instructions (Qiagen, Hilden, Germany). RNA Sample QC, DNase treatment, library preparations, sequencing reactions, and read mapping and alignment were conducted at GENEWIZ, LLC. (South Plainfield, NJ, USA) and are described in the Supplemental Information. After extraction of gene hit counts, the gene hit counts table was used for downstreaming differential expression analysis. Using DESeq2, a comparison of gene expression between the groups of samples was performed. The Wald test was used to generate *p*-values and Log2 fold changes. Genes with adjusted *p*-values < 0.05 and absolute log2 fold changes > 1 were called as differentially expressed genes for each comparison. Significantly differentially expressed genes were used for Gene Set Enrichment Analysis (GSEA) using the fgsea package in the R statistical software (v4.0). Gene sets (pathways) used were downloaded from the Broad Molecular Signature Database and only pathways from Gene Ontology (GO), Reactome, or KEGG were used.

### 2.8. Proliferation Assay In Vitro

Tumor cells were cultured in their respective culture media. On day 0, 200,000 or 1,000,000 cells were seeded in 6-well plates in 3 mL DMEM media (25 mM glucose, 4 mM glutamine, 10% FCS and penicillin/streptomycin). The proliferation of cells was tracked over 72–96 h. At each timepoint, cells were collected via trypsinization and counted using a Countess Automated Cell Counter (Thermo Fisher Scientific, Waltham, MA, USA).

### 2.9. Animal Models

The animal protocol was approved by the Institutional Animal Care and Use Committee of Memorial Sloan Kettering Cancer Center (protocol number: 08-07-011; Approval Date for data presented: 19 September 2014). To develop an orthotopic i.c. tumor model, 200,000 cells suspended in 2 μL of PBS were injected using a 30-gauge needle syringe into the right frontal cortex [stereotactic coordinates: bregma +1.7 mm (anterior), lateral −0.5 mm (right), and at a depth of 2.5 mm] of immunocompetent C57BL/6 female (6–7 weeks old) mice (Charles River Laboratories, Wilmington, MA, USA) and immunocompromised Hsd: Athymic Nude Foxn/nu female (6–7 weeks old) (Envigo, Indianapolis, IN, USA). Animal health was monitored by weighing mice at regular intervals. Intracranial tumor volume (*V*) was calculated from MRI measurements (described later). Kaplan–Meier survival curves were generated, and mean survival time calculated in analysis of Prism GraphPad. The blood samples were collected by retro-orbital venipuncture using standard heparinized microhematocrit capillary tubes. Samples were used to assess LDH enzyme activity in red blood cells. Mice bearing i.c. gliomas were euthanized for H&E, IHC staining when they became lethargic and were noted to have weight loss.

### 2.10. Alzet Pump

A subcutaneous Alzet pump (Model 1007D, Durect Corporation, Cupertino, CA, USA) with an infusion rate 0.5 µL/h and an infusion duration of 7 days was implanted on the back mice, following the MSKCC IACUC protocol. Two groups of mice (treated and control) were established, and both groups were implanted with the Alzet pumps. In the treated group of mice, the Alzet pumps were loaded with 18 mg GNE-R-140 in 100 µL of 100% DMSO, delivering 100 mg/kg/day to ensure a blood concentration of 10 mM over the duration of the experiment. In the control group, the Alzet pumps were loaded with DMSO only.

### 2.11. MR Imaging

Intracranial tumors were imaged using T2-weighted MR imaging; 3 mice were randomly picked up from each group. MRI was performed on a Bruker AV NEO 9.4T scanner equipped with a high-power ID 115 mm gradient capable of a maximum strength of 640 mT/m. An ID 40 mm Bruker quadrature volume coil was used for both RF excitation and detection. The mouse was anesthetized by 2% isoflurane in air, and mouse breathing was monitored by a small-animal physiological monitoring unit. Tumor volume (*V*) was calculated by *V* = (π/6) × long diameter × short diameter × height, which were measured based on MR imaging.

### 2.12. Histological Staining and Image Analysis

Excised tumors were processed with 4% paraformaldehyde followed by paraffin embedding for H&E histology and immunohistochemistry (IHC) studies. IHC staining was performed by the Molecular Cytology Core Facility (MCCF) of MSKCC, using a Discovery XT processor (Ventana Medical Systems, Roche—AZ), in accordance with their established protocols. After 32 min of heat and CC1 (Cell Conditioning 1, Ventana cat#950–500) retrieval, the tissue sections were blocked first for 30 min in Background Blocking reagent (Innovex, catalog#: NB306). The incubation with the primary antibody was performed for 6 h, followed by 60 min incubation with biotinylated goat antirabbit IgG (Vector labs, cat#:PK6101) in 5.75 μg/mL. Blocker D, Streptavidin-HRP and DAB detection kit (Ventana Medical Systems) were used according to the manufacturer instructions. Primary antibodies for LDH-A were obtained from Cell Signaling (#2012), and for LDH-B from Proteintech (#14824-1-AP). The optimal concentration of the primary antibody was determined to be 0.2 µg/mL for LDH-A and 2.0 µg/mL for LDH-B. The slides were counterstained with hematoxylin and cover-slipped with Permount (Fisher Scientific). Quantification of the LDH-A and LDH-B stained sections was performed using FIJI and trainable Weka Segmentation (Image J segmentation plugin) [17,18].

### 2.13. Statistical Analysis

Results are presented as mean ± standard error. Statistical significance was determined by a two-tailed Student *t*-test. A *p*-value of <0.05 was considered significant. All data presented were analyzed using GraphPad Prism (version 7.0; GraphPad Software) and are presented as mean +/− SEM/SD.

## 3. Results

Three different murine glioma cell lines (GL261, CT2A and ALTS1C1) were studied to understand the effects of LDH-A depletion on the growth of cells in culture and in the brain. We have described the variability of effects of LDH-A KD on the three glioma cell lines, including differences in their glycolytic and oxidative metabolic profiles, and subcutaneous tumor-growth characteristics in immunocompetent vs. incompetent host animals (accompanying manuscript [17]). Here we focus on the effects of LDH-A depletion, both genetic (LDH-A shRNA) and pharmacologic (GNE-R-140), on i.c. tumor growth and animal survival, as well as the effects on nutrient depletion on tumor-cell metabolism and phenotype, by measuring changes in cellular bioenergetics by Seahorse Real-Time cell metabolic analysis. A comparison was performed between a genetic approach (using shRNA for LDH-A knockdown (KD)) with a pharmacologic drug-targeted approach that inhibited LDH-A/B enzymatic activity (using GNE-R-140), in the GL261 and CT2A i.c. tumor models. The GL261 and CT2A tumor models were chosen for a direct comparison in this series of experiments, since these cell lines have significantly different LDH-B/LDH-A ratios at both the mRNA and protein level (accompanying manuscript [17]).

### 3.1. LDH-A Knockdown: Effects on Animal Survival and Tumor Growth

**Figure 1:**
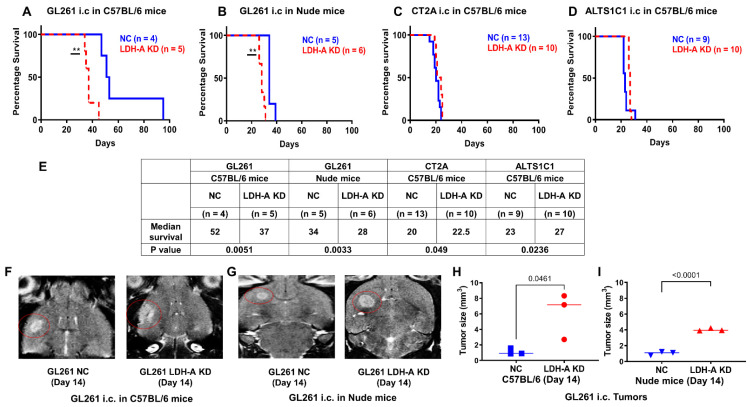
Survival and MR imaging of mice bearing i.c. murine gliomas. Kaplan–Meier survival plots of mice bearing i.c. GL261 (**A**,**B**), CT2A (**C**) or ALTS1C1 (**D**) LDH-A KD and control NC tumors; significant differences are indicated by ** *p* < 0.01. Median survival and comparisons between LDH-A KD and NC tumors (**E**). Representative T2-weighted MR images of i.c. GL261 NC and GL261 LDH-A KD tumors in C57BL/6 mice (**F**) and nude mice (**G**). GL261 tumor volume was estimated on indicated days, using 3-D measurements of the T2-weighted tumor signal in the images; Mean, ±SEM, *n* = 3 (**H**,**I**).

LDH-A KD and control NC glioma GL261, CT2A and ALTS1C1 cells were stereotactically injected into the brain (frontal lobe) of mice. The survival of mice as well as the growth of tumors were monitored using T2-weighted MR imaging. All i.c. tumors grew in immunocompetent C57BL/6 mice after implantation, which differed from the growth of s.c. located GL261 tumors (accompanying manuscript [17]). Unexpectedly, i.c. GL261 LDH-A KD tumors presented a more aggressive phenotype than control GL261 NC tumors, in both immunocompetent (C57BL/6) as well as immunocompromised (nude) mice (Figure 1A,B,E). In contrast, CT2A and ALTS1C1 LDH-A KD tumors were very slightly less aggressive than their control NC counterparts (Figure 1C–E). This result is reflected in the median survival time of tumor-bearing animals estimated from the Kaplan–Meier plots (Figure 1E). The differences in animal survival were consistent with the intracranial size of the tumors, as visualized by MRI in both C57BL/6 (Figure 1F,H) and nude (Figure 1G,I) mice. The slightly longer survival times of LDH-A KD CT2A and ALTS1C1 tumors compared to NC controls is also consistent with our previous findings with 4T1 breast and MyC-CaP prostate tumors [18,19].

### 3.2. LDH Isoenzyme Pattern of i.c. Murine Gliomas

**Figure 2:**
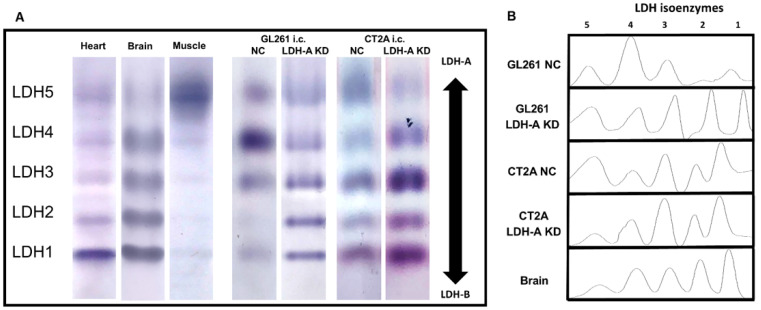
LDH zymograms of i.c. GL261 and CT2A tumors. Native-polyacrylamide gel electrophoretic patterns of i.c. GL261 and CT2A tumors (both LDH-A KD and control NC), as well as heart, brain, and skeletal muscle were obtained (**A**), corresponding LDH isoform analysis (**B**); *n* = 5 independent studies.

To further explore the surprisingly rapid growth of i.c. GL261 LDH-A KD tumors, we performed LDH zymography on the intracranial tumors. Tumors were resected and the LDH isoenzyme profiles of GL261 and CT2A tumors (with and without LDH-A shRNA KD) were compared to each other, and to brain, heart, and skeletal muscle tissue from the same animals (Figure 2). The i.c. GL261 LDH-A KD tumor profile is similar to that of normal brain and heart (LDH-B dominant), and similar to that observed in s.c. tumors [17] (accompanying manuscript [17]). In contrast, i.c. GL261 NC tumors show a LDH4 dominant profile, with modest levels of LDH3 and LDH5. Furthermore, the isoenzyme profile of i.c. GL261 NC tumors is different from that observed in s.c. GL261 NC tumors, where a LDH-A dominant profile was observed (accompanying manuscript [18]). The i.c. CT2A NC and LDH-A KD isoenzyme profiles were similar to each other, but different from the GL261 tumors, with an LDH3 and LDH1 dominant pattern, possibly reflecting some brain tissue contamination. These results indicate that the isoenzyme pattern of tumors can be modulated and may depend on tumor location and the microenvironment (TME), and thereby affect LDH enzyme activity.

### 3.3. Overexpression of Lipid Metabolism Genes in LDHA KD Glioma Cells

**Figure 3:**
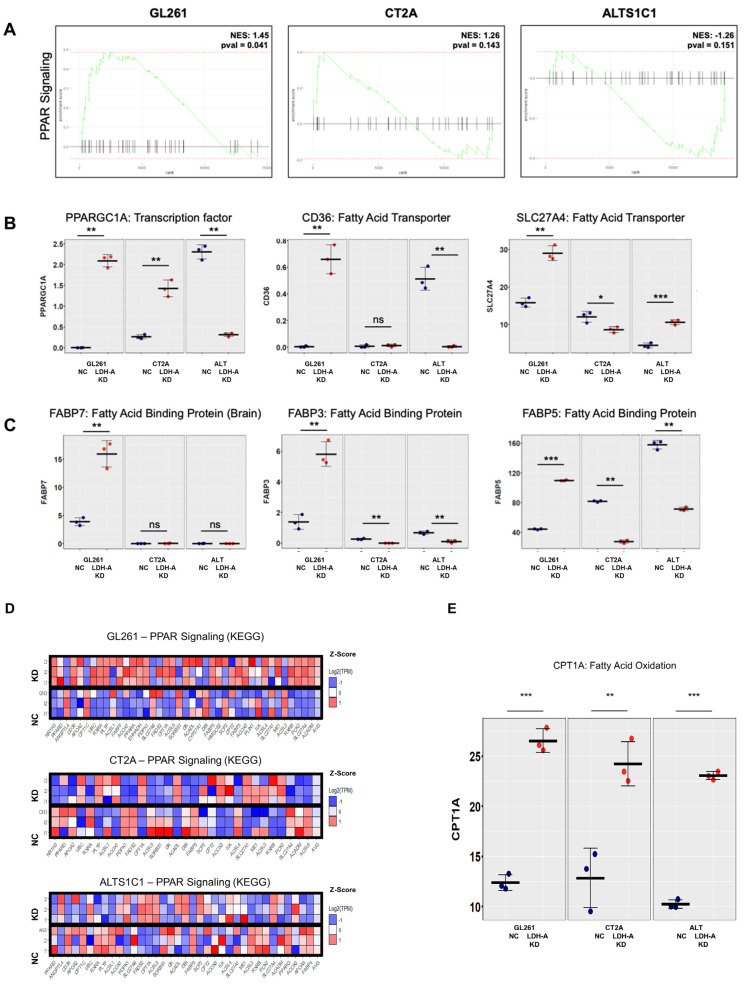
Overexpression of genes involved in lipid metabolism in GL261 LDH-A KD cells. A Gene Set Enrichment Analysis (GSEA) for a single pathway (Peroxisome proliferator-activated receptor (PPAR) pathway) is shown for each GBM cell line (**A**). Transcripts per million (TPM) expression values were plotted in individual cell lines for genes directly involved in PPAR pathway, showing significant upregulation of these genes in (**B**). The Z-transformed scores of individual genes within the PPAR pathway were plotted across each GBM cell line (GL261, CT2A and ALTS1C1). The experiment performed with triplicates, with rows representing each sample, and columns representing individual genes, shows the greatest enrichment in GL261 LDH-A KD cells (**C**). Expression (TPM) of carnitine palmitoyltransferase I (CPT1A) is shown for each cell line (**D**). CPT1A is a mitochondrial enzyme that allows for subsequent movement of acyl carnitine from the cytosol into the intermembrane space of mitochondria, playing an important role in fatty-acid metabolism. Significant differences are indicated by: * *p* < 0.05, ** *p* < 0.01, and *** *p* < 0.001. (**D**,**E**) Overexpression of genes involved in lipid metabolism (PPAR signaling pathway) in LDHA KD glioma cells. Table S4 of the Supplement provides the semi-raw data from the RNASeq analysis that was used to generate the figures.

We performed RNA sequencing (RNASeq) to analyze the cellular transcriptome in all six cell lines (GL261, CT2A and ALT; both scrambled NC and LDH-A shRNA KD) to investigate changes in their expression patterns to define the metabolic pathways upregulated in cells. We confirmed LDH-A depletion in our LDH-A KD cell lines and documented that there was a significantly greater enrichment of oxidative phosphorylation pathway genes (at the mRNA level) in GL261 LDH-A KD cells compared to the other cell lines (accompanying manuscript [17,18]. Here we show an upregulation of fatty-acid catabolism genes, specifically in GL261 LDH-A depleted cells (compared to CT2A or ALT KD cells) (Figure 3). We performed Gene Set Enrichment Analysis (GSEA) from the Kyoto Encyclopedia of Genes and Genomes (KEGG) for lipid and fatty-acid metabolism pathways in each cell line. The analysis revealed a significant enrichment of Peroxisome Proliferator-Activated Receptor (PPAR) signaling pathway genes in GL261 LDH-A KD cells (Figure 3A). The PPAR signaling pathway involves a receptor-activated nuclear transcription factor superfamily that is activated by a diverse number of ligands, and it functions as a transcriptional regulator of many biological processes, including lipid/fatty-acid transport and metabolism, cell proliferation, differentiation, apoptosis, and inflammatory responses, among others. Specific PPAR pathway gene enhancements include: (i) a master regulator of lipid metabolism (PPARGC1A; also known as PGC1-alpha), (ii) lipid transporters (CD36 and SLC27A4) and (iii) intracellular lipid-binding proteins (FABP7/3/5); they were significantly and consistently upregulated in GL261 LDH-A KD cells (Figure 3B). Z-transformed scores of individual genes within the PPAR pathway were plotted across each GBM cell line (GL261, CT2A, and ALTS1C1), showing the greatest enrichment of PPAR pathway genes in GL261 LDH-A KD cells (Figure 3C). Expression (TPM) of a key mitochondrial enzyme involved in fatty-acid metabolism (a component of the PPAR pathway activity)—carnitine palmitoyltransferase I (CPT1A)—is shown for each cell line (Figure 3D). These patterns strongly suggest that GL261 LDH-A depleted cells may be able to utilize lactate and/or fatty acids to support their TCA cycle and increased oxidative phosphorylation.

### 3.4. LDH-A and LDH-B Staining Pattern GL261 and CT2A the Intracranial Tumors

**Figure 4:**
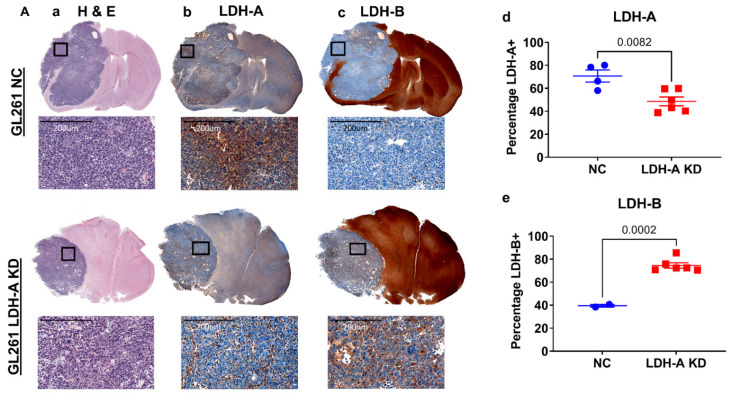

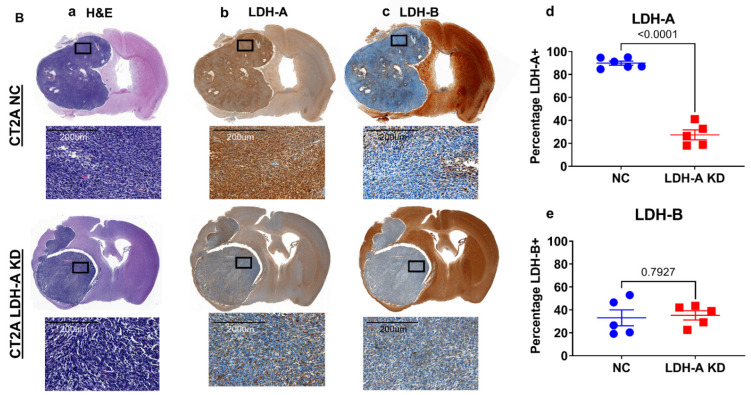
LDH-A and LDH-B immunohistochemistry of i.c. GL261 and CT2A gliomas. H&E staining for GL261 (**Aa**) and CT2A (**Ba**) i.c. tumors (both NC and LDH-A KD); corresponding LDH-A (**Ab**,**Bb**) and LDH-B (**Ac**,**Bc**) immunohistochemistry. The 4 sets of i.c. tumors were grown in immunocompetent C57BL/6 mice. Quantification of percentage of tumor-cell staining (Weka segmentation analysis) for LDH-A (**Ad**,**Bd**) and LDH-B (**Ae**,**Be**) is shown in Ad, Ae, Bd, and Be; mean ± SEM.

In order to better understand how the interplay between LDH-A and LDH-B subunits in the isoenzyme tumor profiles affect the distribution of the proteins in i.c. tumors, we performed immunohistochemistry, with specific staining for LDH-A and LDH-B. H&E staining showed a relatively homogenous pattern for both i.c. GL261 and CT2A tumors, with little necrosis or stroma, and minimal infiltration of adjacent brain tissue (Figure 4(Aa,Ba), left column). LDH-A and LDH-B immunohistochemistry showed a more variable pattern of staining for both tumors (Figure 4(Ab,Ac,Bb,Bc); middle two columns). A Weka analysis showed significantly greater LDH-A staining in GL261 NC and CT2A NC tumors compared to the LDH-A KD tumors (Figure 4(Ad,Bd), far right graphs; Table S1). In contrast, a significantly greater LDH-B staining pattern was observed in GL261 LDH-A KD tumors (compared to the NC tumors), whereas there was no LDH-B staining difference between CT2A LDH-A KD and NC tumors (Figure 4(Ae,Be), far-right graphs; Table S1). These results are consistent with the isoenzyme profiles (Figure 2) and they confirm (i) downregulation of LDH-A expression in i.c. GL261 and CT2A LDH-A KD tumors compared to the NC controls, and (ii) upregulation of LDH-B expression in i.c. GL261 LDH-A KD tumors, but not in CT2A LDH-A KD tumors.

In many—but not all—GL261 i.c. tumor regions, there was an inverse relationship between LDH-A and LDH-B staining intensity (Figure S1A). This inverse relationship was greater for LDH-A KD than NC GL261 tumors. CT2A tumors showed a different relationship; there was a more direct relationship between LDH-A and LDH-B staining intensity (Figure S1B). We have also described similar relationships in s.c. GL261 and CT2A tumors (accompanying manuscript [17]). Brain tissue stained strongly for LDH-B and weakly for LDH-A, particularly in grey-matter regions (Figure 4(Ab,Ac,Bb,Bc)), which is also consistent with the isoenzyme profile analysis (Figure 2). Furthermore, there appeared to be a rough inverse relationship in staining intensity between grey and white matter regions, where white matter stained more intensely for LDH-A than grey matter. These results demonstrate a considerable intratumoral variability of LDH-A and LDH-B expression and suggests that different cell types have distinct metabolic profiles [21,22].

### 3.5. Impact of Nutrients on Tumor-Cell Metabolism and Proliferation

We characterized the bioenergetics profile (ECAR and OCR) of GL261 NC and LDH-A KD cells in more detail under different nutrient conditions. Given our RNASeq results for GL261 LDHA-depleted cells showing the unique upregulation of LDH-B (accompanying manuscript [17,18] we focused on whether these cells may be better able to metabolize lactate to pyruvate.

First, we investigated the influence of the absence of glucose and pyruvate on the major bioenergetic parameters. Not surprising, ECAR was significantly lower in absence of glucose (compared to the presence of 10 mM glucose), and significantly lower in KD than NC cells under both conditions (Figure S2A,C, Table S2). The presence or absence of pyruvate had little or no effect on ECAR (Figure S3A,B); a very slow or no decrease in ECAR was observed over 6 h of incubation in both NC and KD GL261 cells (Figure S3A,B). In the absence of glucose (with or without pyruvate) in the media, OCR values were significantly higher in both NC and LDH-A KD GL261 cells, (Figure S2B,D, Table S2), when compared to basal OCR in the presence of 10 mM glucose (accompanying manuscript [17,18] OCR values for both KD and NC GL261 cells were comparatively stable over 6 h of incubation in both the presence and absence of pyruvate (Figure S3C,D). These results suggest that exogenous pyruvate is not a major carbon source for metabolism through the TCA cycle for GL261 NC or LDH-A KD cells.

Second, we explored the impact of adding lactate to the incubation medium on bioenergetic parameters (Seahorse metabolic experiments). The effect of adding 10 mM Na-lactate to the incubation medium that lacked glucose (but contained 2 mM glutamine), both in the presence and absence of 1 mM pyruvate, was also studied in GL261 NC and LDH-A KD cells. ECAR dramatically decreased in the absence of glucose, before and after the addition of 10 mM Na-lactate in both GL261 NC and LDH-A KD cells; all four experimental sets had similar profiles (Figure S4A,C; Table S3). In contrast, OCR showed a markedly different pattern than ECAR. Control NC cells were able to maintain stable oxidative phosphorylation (OCR) in the presence of 10 mM lactate and 1 mM pyruvate (in the absence of glucose), but not in the absence of both pyruvate and glucose (Figure S4B,D; Table S3). OCR in NC cells was highly dependent on pyruvate in the media (in the absence of glucose), suggesting that exogenous pyruvate (in the presence of 2 mM glutamine) can support oxidative phosphorylation in GL261 NC cells (Figure S4B,D; Table S3). In contrast, OCR gradually increased over the 360 min time course in GL261 KD cells with 10 mM Na-lactate added to the media, and this increase was not dependent on pyruvate (Figure S4B,D; Table S3). These results clearly demonstrate that lactate alone (in the absence of glucose and pyruvate, but in the presence of 2 mM glutamine) can support effective OCR (OXPHOS) in GL261 KD cells, confirming RNA-seq data that exogenous lactate may be functioning as a metabolic energy source, as well as a signaling molecule [3,23]. These results clearly demonstrate that GL261 KD cells (but not NC cells) can effectively metabolize lactate as a primary carbon source through the Kreb’s cycle (TCA) [24], and that OCR is further enhanced by the presence of 1 mM pyruvate in the media. Lactate added to the media is likely transported into the cell and metabolized through pyruvate (facilitated by high LDH-B expression), prior to entering the Kreb’s cycle. These results also suggest that GL261 LDH-A KD cells changed they properties regarding substrate utilization, which may be a linked with the changes in the LDH-B/LDHA ratio that occur following LDH-A KD. The effect of 2 mM glutamine (compared to the absence of glutamine) in the media on ECAR and OCR was not part of the above studies.

**Figure 5:**
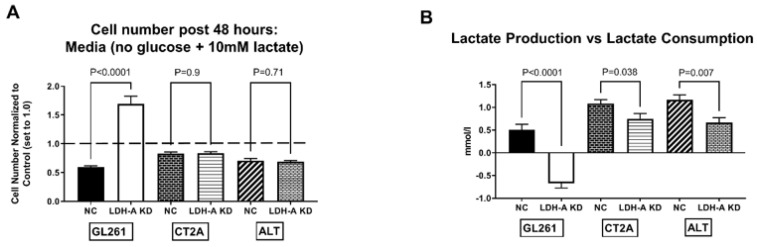
Effect of Na-lactate on the proliferation and the production vs. consumption of lactate in LDH-A KD vs. control NC glioma cell lines. Effect of adding 10 mM Na-lactate to the culture media containing 0 mM Glucose on the 48 h proliferation of GL261, CT2A and ALTS1C1 murine glioma cell lines (comparing LDH-A KD to the NC control; 6 cell lines) (**A**). The control measure of proliferation (cell number after 48 h of incubation) in the absence of both lactate and glucose is set to 1.0, (dashed line); the experiment addresses whether Na-lactate alone can support cell proliferation (**A**). Lactate concentration was measured in the culture media before and after the 48 h incubation (**B**); the starting lactate concentration (0 h) was subtracted from the final concentration (48 h), and the difference is plotted (**B**). A positive value indicates “lactate production”, whereas a negative value indicates “lactate consumption” (**B**). Values are mean, ±SEM; *n* = 3.

Third, the influence of 10 mM Na-lactate on cell proliferation was also investigated, to study the effect of major nutrient (glucose and pyruvate) deprivation vs. availability. The proliferation of GL261 LDH-A KD cells in culture was significantly slower than the NC control cells in standard media (25 mM glucose 4 mM glutamine and 0 mM pyruvate) (Figure S5A). No significant difference was observed between CT2A and ALTS1C1 KD and NC cells) (Figure S5B,C). The addition of 10 mM Na-lactate significantly decreased the proliferation of all NC cell lines, as well as the proliferation of CT2A and ALTS1C1 LDH-A KD cells. Interestingly, only the proliferation of the GL261 LDH-A KD cell line was unaffected by the addition of 10 mM Na-lactate (Figure S5A). To further assess the ability of cells to metabolize lactate, GL261, CT2A and ALTS1C1 KD and NC cells (6 experimental sets) were incubated in glucose-free standard media (see above), with and without 10 mM Na-lactate. Cell proliferation was significantly reduced in the absence of glucose in the media for all six cell lines (data not shown; p values were: GL261: NC, *p* = 0.007; LDH-A KD, *p* = 0.04. CT2A: NC, *p* = 0.001; LDH-A KD, *p* = 0.003. ALTS1C1: NC, *p* = 0.006; LDH-A KD, *p* = 0.086). Interestingly, only the proliferation rate of GL261 KD cells showed a significant increase (*p* < 0.0001) following the addition of 10 mM Na-lactate to the glucose-free media (Figure 5A). All other cell lines showed a further decrease in the proliferation rate following 10 mM Na-lactate addition, particularly GL261 NC cells (Figure 5A).

Fourth, lactate production and consumption were evaluated under the same growth conditions noted above. Lactate was measured in the media before and after a 48 h incubation of the six experimental cell lines (Figure 5B). Subtracting the preincubation lactate measurement from the post-48 h incubation lactate measurement demonstrated whether there was net lactate production (+value) or consumption (−value) (Figure 5B). Again, only GL261 KD cells showed a “consumption” of lactate during the 48 h incubation period, consistent with LDH-B dominance of its isoenzyme profile upon the LDH-A downregulation. All the other cell lines (including GL261 NC) showed a net “production” of lactate, consistent with LDH-A dominance. As expected, all the NC cells produced more lactate than the KD cells. These results further suggest that despite lactate being recognized as a waste product in the past, it can be used by variety of cancer cells for OXPHOS and in combination with glycolysis for ATP production [3]. Furthermore, GL261 NC cells produced less lactate than CT2A and ALTS1C1 NC cells (Figure 5B), which is also consistent with the lower expression of LDH-A in GL261 cells compared to that in CT2A and ALTS1C1 cells (Figure 5A and accompanying manuscript [17,18]).

### 3.6. GNE-R-140: Effects on GL261, CT2A Glioma Cells in Culture

**Figure 6:**
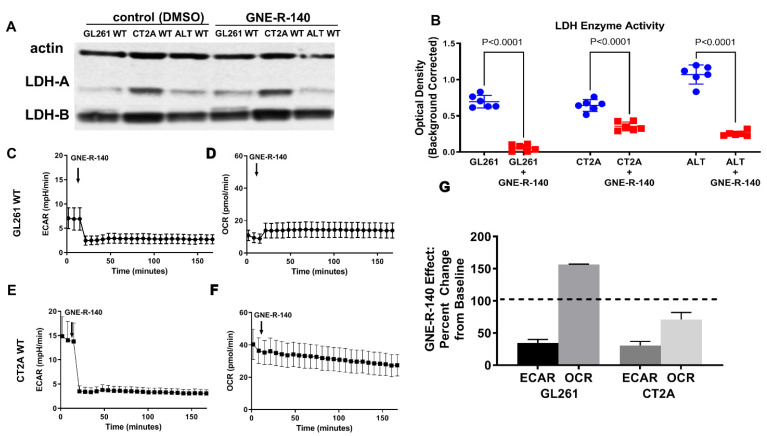
GNE-R-140 effects on GL261, CT2A and ALTS1C1 wild-type cells. Western blot expression of LDH-A and LDH-B proteins, in the presence of vehicle (DMSO) and drug (GNE-R-140, 10 µM) for WT murine glioma cells (**A**). Effect of GNE-R-140 (10 µM) on LDH enzyme activity of WT murine glioma cells (**B**). Effect of adding GNE-R-140 (10 µM) to the medium on ECAR—glycolysis (**C**,**E**) and OCR (OXYPHOS) (**D**,**F**) of GL261 WT cells (**C**,**D**), and of CT2A WT cells (**E**,**F**), respectively. Panel (**G**) shows the percentage change over 150 min in ECAR and OCR from baseline (100%) for GL261 and CT2A WT cells, when treated with GNE-R-140. Mean ± SEM. The native Western blot for Panel A is shown in the Supplement (Figure S7).

GNE-R-140 is a potent LDH inhibitor that can modulate LDH-A activity both in vitro and in vivo [25]. To further understand the mechanism of action in wild-type glioma cells, we evaluated the influence of the drug on LDH-A and LDH-B protein expression and LDH enzyme activity. GNE-R-140 (10 µM) had no effect on LDH-A or LDH-B protein levels, as observed in Western blot (Figure 6A), but significantly inhibited LDH enzymatic activity of the GL261, CT2A and ALTS1C1 wild-type cell lines (Figure 6B), with a more profound effect on the LDH enzyme activity in GL261 cells. The addition GNE-R-140 (10 µM) during a Seahorse assay produced an acute decrease in ECAR of GL261 and CT2A wild-type cells (Figure 6C,E,G). In contrast, we noticed that OCR increased only in GL261 cells, which remained stable over 150 min (Figure 6D,G). Meanwhile, OCR decreased slowly in CT2A cells over the same time period (Figure 6F,G). GNE-R-140 (10 µM) also significantly reduced cell proliferation of all three wild-type cell lines (Figure S6A). Moreover, as we have seen before, the proliferation of GL261 cells was slower compared with other cell lines (accompanying manuscript [18]). In the presence and absence of added Na-lactate (10 mM), GNE-R-140 (10 µM) reduced intracellular lactate concentrations in wild-type GL261 cells in culture (Figure S6B), similar to that of LDH-A KD (compared to NC) in the LDH-A KD cell lines (Figure S6C). These results suggest that GNE-R-140 is affecting cells with lower levels of: (a) LDH-A mRNA and protein expression, (b) LDH enzymatic activity, and (c) proliferation (e.g., GL261), more than cells with higher levels of LDH-A expression and proliferation (accompanying manuscript [17]).

### 3.7. GNE-R-140: Effects on i.c. GL261, CT2A Tumors

**Figure 7:**
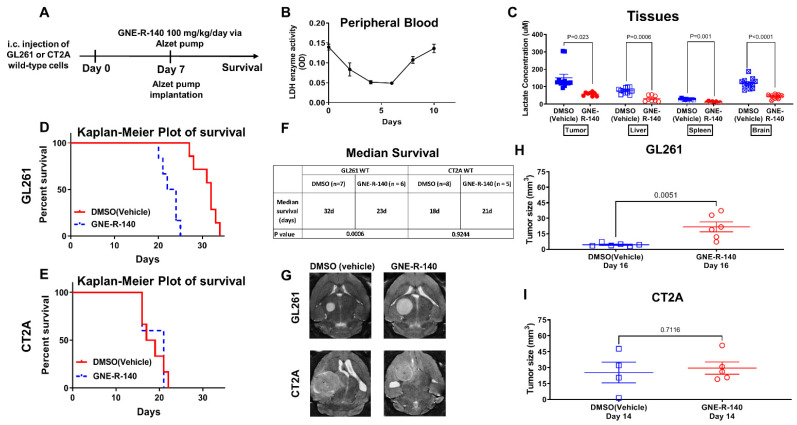
GNE-R-140 treatment effects on GL261 and CT2A i.c. tumors. Outline of the experimental protocol for evaluating the LDHA inhibitor (GNE-R-140) (**A**). Time course of GNE-R-140 treatment effect on venous blood (RBC) LDH enzyme activity (**B**). Ex vivo intracellular lactate concentration from GL261 tumors and tissue extracted at day 10, comparing GNE-R-140 treatment vs. vehicle (DMSO) control treatment (**C**). Kaplan–Meier plots of animal survival following i.c. implantation of WT GL261 and CT2A tumors and treatment (**D**,**E**). Median animal survival time is shown in the table (**F**). Representative T2-weighted MRI images of GL261 tumors (at day 16 post-implantation), and CT2A tumors (at day 14 post-implantation); 7 days after initiating GNE-R140 treatment (**G**). Comparison of MRI-measured tumor size on indicated days for GL261 (**H**) and CT2A (**I**) wild-type tumors following GNE-R-140 and vehicle treatment.

To investigate in vivo activity of GNE-R-140, we developed a protocol where the drug was administered continuously by a s.c. located Alzet pump for 7 days (Figure 7A), in contrast to the previous oral-delivery approach [25]. The time course of the systemic effect of GNE-R-140 on LDH activity in mice was assessed by measuring enzymatic activity of LDH in venous blood (RBCs). LDH activity of RBCs decreased during days 2–6 but returned to baseline level by day 10 (Figure 7B). In addition, lactate production was assessed for different tissues, including GL261 wildtype tumors (Figure 7C). Kaplan–Meyer (KM) plots and MR imaging of i.c. wild-type GL261 and CT2A gliomas (GNE-R-140 vs. DMSO control) were obtained (Figure 7D–I). The KM plots showed a shorter median survival (23 vs. 32 days, *p* = 0.0006) for the GL261 GNE-R-140 treatment group compared to the DMSO-treated controls (Figure 7D,F–H). No treatment effect was observed for the CT2A gliomas (Figure 7D,F,G,I). This is a similar pattern of treatment response observed when comparing the KM plots and MR imaging of animals bearing genetically shRNA modified GL261 and CT2A cells and i.c. tumors (LDH-A KD vs. NC GL261) (Figure 1). GNE-R-140 treatment and LDH-A KD produced similar results: (i) increased i.c. tumor growth, and (ii) reduced the survival of animals bearing i.c. GL261 gliomas, whereas there was little or no effect on i.c. CT2A gliomas. These results further suggest that the unexpected growth properties of GL261 LDH-A KD cells in a brain microenvironment, as well as GNE-R-140 treated wild-type GL261 i.c. tumors. This difference may reflect the higher levels of LDH-B and lower levels of LDH-A expression (and LDH enzyme activity), resulting in significant changes in the activity of specific metabolic pathways, metabolite utilization, and metabolite formation.

## 4. Discussion

Three intracranial murine gliomas were studied and we compared the effects of genetic-shRNA LDH-A knockdown [19,25,26] and LDH drug-targeted inhibition [19,25,26] on tumor-cell metabolism, nutrient dependence, tumor growth, and animal survival time. We show that murine glioma cells (i) can engage different metabolic pathways to support their proliferation; (ii) have a varied dependence on specific nutrients and nutrient combinations (glucose, pyruvate, and lactate); and (iii) have differences with respect to lactate production vs. consumption. We also compared the responses to genetic-shRNA LDH-A knockdown vs. LDH drug-targeted inhibition [19,25,26]. We show that LDH drug-targeted inhibition with GNE-R-140 reproduces the effects of LDH-A shRNA knockdown on tumor-cell metabolism, tumor growth, and animal survival.

The regulation and function of LDH-A and lactate in human gliomas and other solid tumors has gained increased attention over the past decade [27]. Serum LDH and lactate are well-known markers of aggressive systemic tumors, and it was recently shown that serum lactate levels are also associated with the grade of gliomas [28]. It is also known that MiR-200b is a regulator of tumor progression and metabolism targeting LDH-A in human malignant gliomas [29], and that upregulation of KLHDC8A (Kelch domain-containing 8A) is induced by lactate and contributes to the proliferation, migration, and apoptosis of human glioma cells [20]. Furthermore, LDH-A silencing occurs in IDH mutant gliomas, and likely contributes to their characteristically slower progression [30]. These studies (and others) demonstrate the important role of LDH-A and lactate in human gliomas, and suggest that LDH-A may be a potential therapeutic target [27]. However, our studies show that not all murine gliomas are alike, and that the level of LDH-A expression and its interplay with LDH-B can lead to changes in the activity of different metabolic pathways and complex interactions between tumor cells and their environment. These changes and interactions need to be carefully assessed, since the inhibition of glycolysis in tumor cells may lead to undesirable metabolic and phenotypic effects, including increased tumor aggressiveness.

The isoenzyme profiles of i.c. GL261 NC and LDH-A KD tumors were clearly different, whereas the isoenzyme profiles of i.c. CT2A NC and LDH-A KD were similar. Although the LDH-A and LDH-B immunohistochemistry and a Weka analysis were generally consistent with the in vitro metabolic analyses and the LDH zymography, our analyses clearly demonstrate an intratumor variability in the spatial and level of LDH-A and LDH-B expression in these tumors. GL261 LDH-A KD i.c. tumors grew more rapidly and resulted in shorter animal survival times than control i.c. GL261 NC tumors. In contrast, LDH-A KD had little or no effect on the growth of CT2A and ALTS1C1 i.c. tumors. The differences in tumor-cell metabolism, LDH isoenzyme, LDH-A/LDH-B immunohistochemistry, and tumor-growth and animal survival patterns were clearly shown to be related to LDH-A depletion and an associated increase in LDH-B expression. Interestingly, it has been suggested that the sole (or high) expression of LDH-B could identify an important biological marker of glioma cells that is critical for their progression, and it might afford a new target for anticancer drugs [31].

The metabolic patterns, we observed in GL261 LDHA-depleted cells strongly suggest that they are able to utilize lactate and/or fatty acids to support an active TCA cycle and an increase in oxidative phosphorylation (accompanying manuscript [17,18]). Our RNA-seq bioinformatics analysis provides additional evidence that GL261 LDH-A KD cells may have an improved ability to metabolize fatty acids and lipids through enhancement of the PPAR pathway and upregulation of fatty-acid catabolism genes (specifically in GL261 LDH-A depleted cells). The PPAR signaling pathway involves a receptor-activated nuclear transcription factor superfamily that is activated by a diverse number of ligands, and it functions as a transcriptional regulator of many biological processes, including lipid/fatty-acid transport and metabolism, cell proliferation, differentiation, apoptosis, and inflammatory responses, among others. These patterns strongly suggest that GL261 LDH-A depleted cells may be able to utilize lactate and fatty acids to support their TCA cycle and increased oxidative phosphorylation.

These data are consistent with recent observations that show increased mitochondrial biogenesis induced by activation of the CREB-PGC1a pathway, which triggers a metabolic shift and differentiation in glioma cells [32]. Blocking mitochondrial biogenesis by silencing PGC1a abrogates differentiation; conversely, overexpression of PGC1a elicits differentiation, showing that mitochondrial biogenesis and the metabolic switch to oxidative phosphorylation drive the differentiation of tumor cells [32]. Interestingly, others have recently shown a novel carbohydrate-metabolism regulation through protein–metabolite interactions [33]. For example, both ATP accumulated during oxidative phosphorylation and long-chain fatty acetyl-CoAs inhibit LDH-A, but not LDH-B, and long-chain fatty acids caused a loss of pyruvate/lactate interconversion only in cells dependent on LDH-A [33].

It has also been shown that lactate is a primary circulating TCA substrate in many tissues and tumors [34], and that mitochondria play a central and multifunctional role in malignant tumor progression [35]. However, different tumors manage lactate differently; some tumors are lactate producers/excreters and some tumors are lactate consumers/utilizers. Furthermore, within a single heterogenous tumor there may be shuttling of lactate between different cell types [36]. Some cancer cells cannot utilize lactate [37], while other tumors can easily use lactate as a fuel, and respond to supplemental lactate with increased proliferation and vascularity [37]. The detection of labeled lactate in the TME can reflect two processes: (1) exogenous lactate uptake from the circulation or (2) glycolysis-derived lactate (produced by tumor and/or stromal cells), which are dependent on MCTs and LDH, respectively. Tumor perfusion can also be an important factor that influences lactate accumulation from the circulation. If perfusion or MCT activity is limited, then glucose-derived lactate will predominantly accumulate in the tumor, and circulation-derived lactate will be limited. Recently, the infusion [1,2-C^13^] glucose in patients bearing triple-negative breast tumors detected more labeled pyruvate and lactate in tumors than in the circulation, indicating the predominance of locally synthesized lactate from glucose in these highly glycolytic breast tumors [38]. We clearly show that GL261 LDH-A KD cells consume lactate and that 10 mM Na-lactate (in the absence of glucose) supports cell proliferation, whereas this is not the case for GL261 NC and the other cell lines we studied (Figure S4). These results in GL261 LDH-A KD cells (but not in the other cell lines) are consistent with findings that lactate can serves as a carbon source in tumors [34,39], particularly in a nutrient (glucose) deficient TME [39]. Recently, circulating lactate has been suggested as a “universal fuel” [34]. In several mouse models of lung and pancreatic cancers, C^13^-lactate infusion contributed significantly to TCA cycle metabolites [34]. Our in vitro nutrient modulation studies support this hypothesis for GL261 LDH-A KD cells.

Our results do not completely correspond to the findings of Rabinowitz et al. [34,40], who showed that lactate exchange between the tumor and the circulation is rapid via MCT transporters. Our RNA-Seq data show that MCT1 (*SLC16A1*) is lowest in GL261 NC and LDH-A KD cells (accompanying manuscript [17]), compared to the other glioma cell lines. Furthermore, there was a significant reduction in the expression of the lactate exporter MCT4 (*SCL16A3*) only in GL261 LDH-A KD cells. This raises the question as to whether a rapid intra/extracellular exchange of lactate is taking place in these cells and tumors. Furthermore, these findings support our hypothesis that inhibition of glycolysis in LDH-A KD GL261 tumor cells leads to increased fatty-acid catabolism.

Recently, there have been several studies focusing on the targeting of cancer metabolism through genetic knockout (or knockdown) of LDH-A and by using small-molecule inhibitors of LDH-A [25,41,42,43]. In human colon adenocarcinoma and murine melanoma cells, neither *LDH-A* nor *LDH-B* knockout strongly reduced lactate secretion, whereas the double knockout (*LDHA/B*-DKO) fully suppressed LDH activity and lactate secretion [25]. These results were reproduced pharmacologically by treating WT cells with the LDHA/B-specific inhibitor GNE-R-140. Pancreatic cell lines that predominantly utilize oxidative phosphorylation (OXPHOS) rather than glycolysis were inherently resistant to GNE-140, but could be resensitized to GNE-R-140 with the OXPHOS inhibitor phenformin [41]. Acquired resistance to GNE-140 was driven by activation of the AMPK-mTOR-S6K signaling pathway, which led to increased OXPHOS, whereas inhibitors targeting this pathway prevented resistance. Peptides sequences with high affinity to the β-sheet region of LDH-A (LDH5) were shown to inhibit enzymatic activity; a lead peptide (cGmC9) inhibited LDH-A activity in vitro in the low-micromolar range and was more efficient than GNE-R-140 [42]. In addition, the coregulation of LDH and the heat shock response with respect to radiation resistance was presented [43]. It was shown that that inhibition of LDH, either pharmacologically (oxamate or GNE-R-140) or by gene knockout of *LDHA* and *LDHB*, significantly increases the radiosensitivity in tumor cells by a global impairment of the stress response. The authors suggest that inhibition of the lactate metabolism might provide a promising strategy in the future to improve the clinical outcome of patients with highly aggressive, therapy-resistant tumors.

What is clear from our study and those discussed above is that tumors are not all alike, and that the level of LDH-A expression and the interplay with LDH-B can lead to metabolic changes and complex interactions between tumor cells and their environment. These interactions need to be carefully assessed, since the inhibition of glycolysis in tumor cells may lead to undesirable metabolic and phenotypic changes, including increased tumor aggressiveness.

## 5. Conclusions

The effects of genetic-shRNA LDH-A knockdown and LDH drug-targeted inhibition (GNE-R-140) on murine glioma-cell metabolism, nutrient dependence, tumor growth, and animal survival time were similar. Although inhibition of LDH-A/glycolysis may be an attractive strategy to inhibit the growth of some systemic tumors, the level of LDH-A expression and its interplay with LDH-B can lead to complex metabolic interactions between tumor cells and with their environment. These interactions need to be carefully assessed, since the inhibition of glycolysis in tumor cells may lead to metabolic and phenotypic changes resulting in increased lactate, fatty-acid, and lipid catabolism, and increased tumor aggressiveness, as shown in this manuscript. Our study suggests that inhibition of LDH-A/glycolysis may not be a general strategy to inhibit the i.c. growth of gliomas, since the level of LDH-A expression and its interplay with LDH-B can lead to complex metabolic interactions between tumor cells and their environment and result in a more aggressive tumor phenotype.

## Data Availability

Table S4 of the Supplementary Materials provides the semi-raw data from the RNA Seq analysis that was used to generate the figures. The Blasberg Lab has closed at MSKCC, although relevant data exists on the MSK servers that can be accessed by Dr. Blasberg, he currently is an Emeritus Professor.

## Supplementary Materials

The following supporting information can be downloaded at: https://www.mdpi.com/article/10.3390/cancers14092306/s1, Figure S1: Local intratumoral LDH-A vs. LDH-B protein expression relationships in i.c. GL261 and CT2A, NC and LDH-A KD tumors; Figure S2: Effect of injected Na-Lactate on metabolism of NC and LDH-A KD GL261 cells, in the presence and absence of glucose; Figure S3: Metabolism of NC and LDHA KD GL261 cells in the presence and absence of glucose and pyruvate; Figure S4: Effect of injected Na-Lactate on metabolism of NC and LDH-A KD GL261 cells, in the absence of glucose and in the presence or absence of pyruvate; Figure S5: Effect of Na-Lactate on the proliferation of LDH-A KD and control NC glioma cell lines in the presence and absence of glucose; Figure S6: Effect of GNE-R-140 on cellular proliferation and intracellular lactate concentration; Figure S7: The native western blot for Panel A of Figure 6. Table S1: Weka segmentation analysis of IHC staining for LDH-A and LDH-B of i.c. GL261 and CT2A tumors; Table S2: Comparison of ECAR and OCR changes in GL261 cells following administration of 10 mM Na-Lactate to the media, in the presence (Glu+) and absence (Glu-) of 10mM glucose; Table S3: Comparison of ECAR and OCR changes in GL261 cells following administration of 10 mM Na-Lactate to non-Glucose (0 mM) containing media, in the presence (Pyr+) and absence (Pyr-) of 1mM Pyruvate; Table S4: Semi-raw data from the RNASeq analysis that was used to generate Figure 3.
