# Supplementary material for: Genetic and Drug Inhibition of LDH-A: Effects on Murine Gliomas
Source: Cancers (Basel). 2022 May 6;14(9):2306. doi: 10.3390/cancers14092306 (PMC9105502; doi:10.3390/cancers14092306)
Supplement: Supplementary file 1 [file cancers-14-02306-s001.zip › Supplementary Information (File1) - cancers-1518208.pdf]

Table S1: Weka segmentation analysis for IHC Staining for LDH-A and LDH-B of s.c. GL261 and CT2A tumors

	GL261 in C57BL/6 mice		GL261 in nude mice		CT2A in C57BL/6 mice	
	NC	LDH-A KD	NC	LDH-A KD	NC	LDH-A KD
LDH-A+ % of tumor	70.7± 5.2	48.6 ± 3.8	78.5 ± 2.9	34.5 ± 2.0	89.93 ± 4.4	27.38 ± 9.7
P value	0.0082		<0.0001		<0.0001	
LDH-B+ % of tumor	39.6 ± 1.17	74.5 ± 2.31	38.8 ± 3.48	78.6 ± 4.57	33.1 ± 15.6	35.29 ± 9.0
P value	0.0002		<0.0001		0.79	

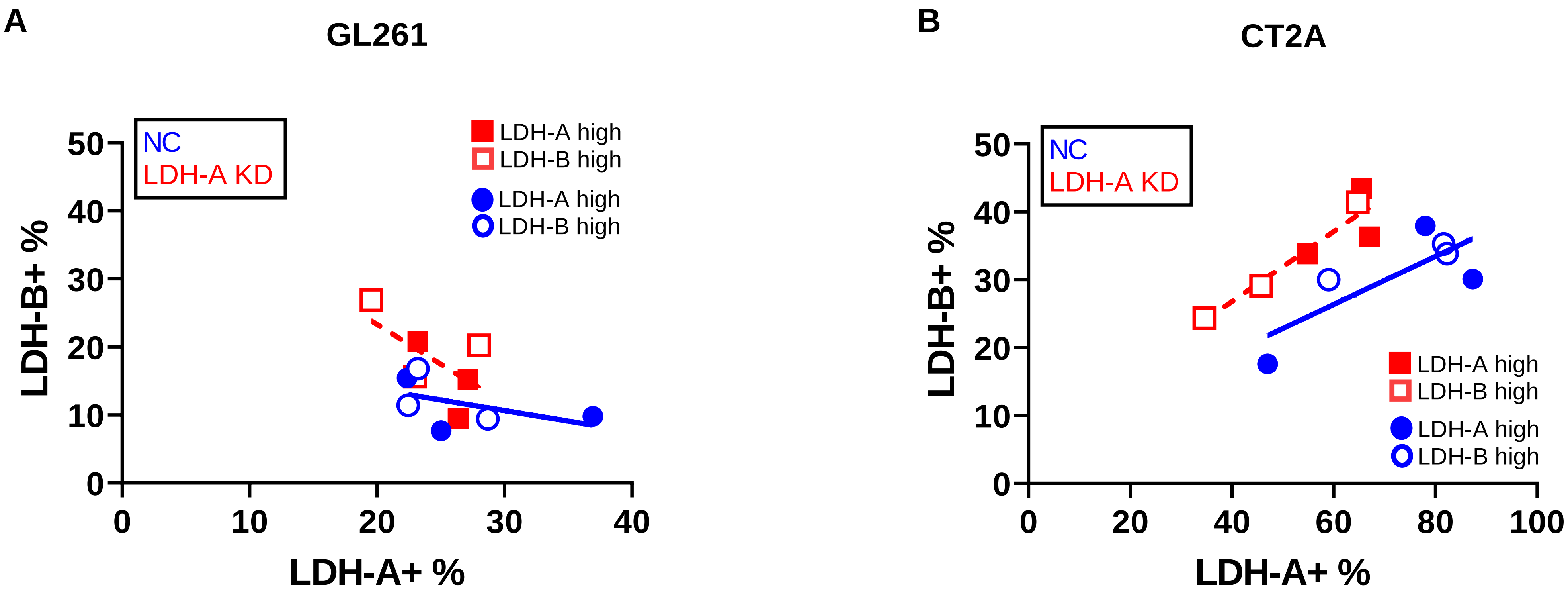

Figure S1. LDH-A and LDH-B correlation.

LDH-A and LDH-B correlation in GL261 and CT2A intracranial gliomas. Local intratumoral LDH-A and LDH-B staining relationships for i.c. GL261 and CT2A tumors. The percentage of tumor cells staining positive for LDH-B was plotted vs the percentage of cells staining positive for LDH-A in both high LDH-B+ cell density regions (solid symbols) and high LDH-A+ cell density regions (open symbols). A local "inverse" intratumoral LDH-A and LDH-B staining intensity relationship for i.c. GL261 tumors **(A),** and "direct" staining intensity relationship for i.c. CT2A tumors **(B).**

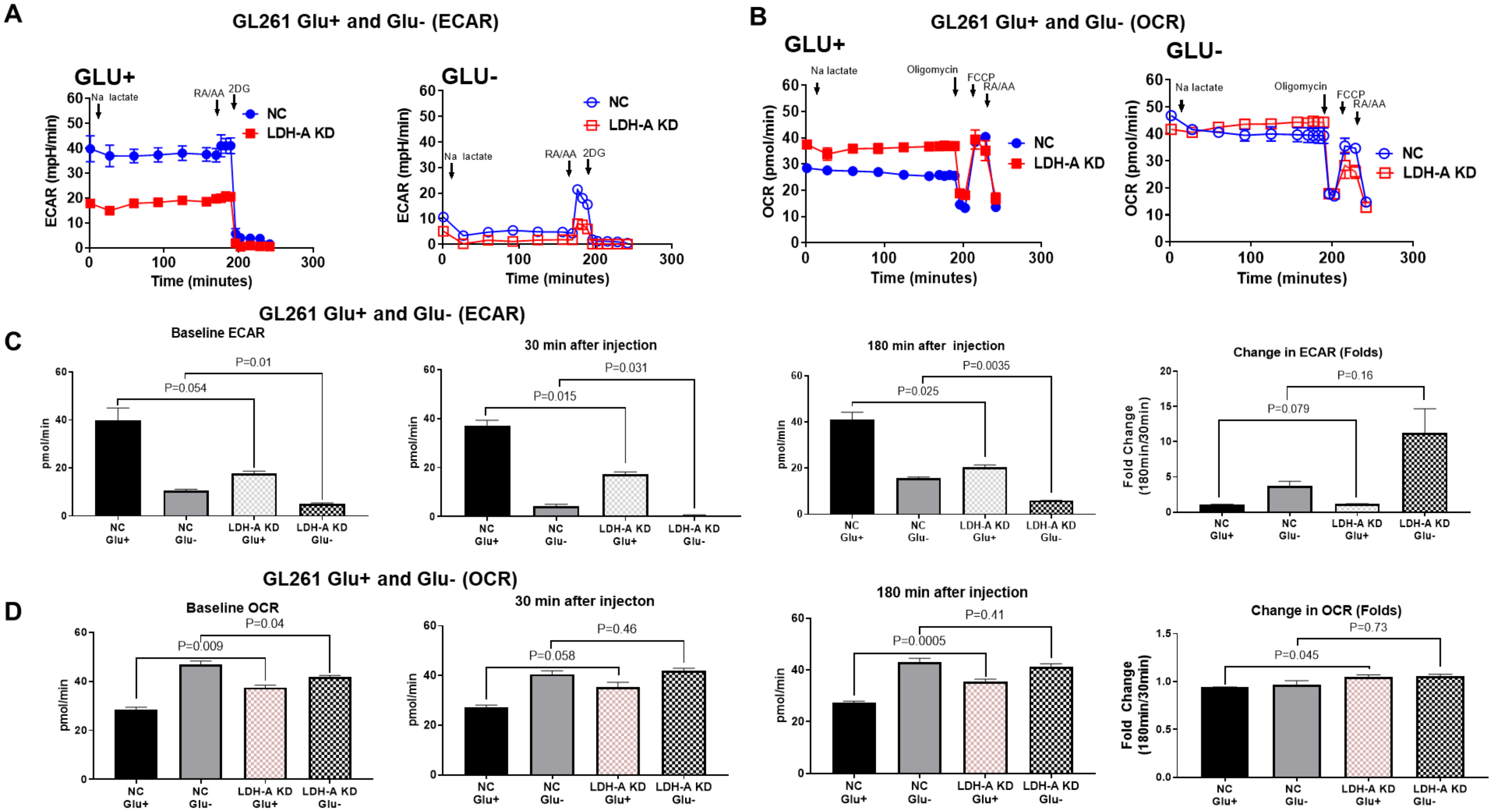

Figure S2. Effect of injected Na-Lactate on metabolism of NC and LDH-A KD GL261 cells, in the presence and absence of glucose. Real time profile of the Extracellular Acidification Rate (ECAR) (**A**) and Oxidative Phosphorylation Rate (OCR) (**B**) in NC and LDH-A KD GL261 cells, following the addition of Na-Lactate to the media, in presence (Glu+) and absence (Glu-) of 10mM glucose. Observed difference in Baseline ECAR (**C**) and OCR (**D**), as well as at 30 min and at 180 min values following the addition of Na-Lactate to the media. Statistical significance of differences between NC and LDH-A KD cells, both in the presence and absence of 10mM glucose were assessed (summarized in Table S-2). Far right panels depict the changes in ECAR and OCR between 30 and 180 minutes (summarized in Table S-2). Values are mean, ±SEM; n = 2.

Table S2: Comparison of ECAR and OCR changes in GL261 cells following administration of 10 mM Na-Lactate to the media, in the presence (Glu+) and absence (Glu-) of 10mM glucose

	GL261 cell line	P values for comparisons between groups	
		NC Glu+ vs NC Glu-	LDH-A KD Glu+ vs LDH-A KD Glu-
ECAR	Baseline ECAR	0.031	0.005
	30 min after injection	0.005	0.004
	180 min after injection	0.016	0.004
	Change in ECAR 30-180 min (Folds)	0.063	0.097
OCR	Baseline OCR	0.003	0.026
	30 min after injection	0.006	0.044
	180 min after injection	<0.0001	0.008
	Change in OCR 30-180 min (Folds)	0.68	0.73

See Figure S4. Basic media contained DMEM without serum, phenol red, sodium bicarbonate. 10mM glucose, 10mM Na pyruvate, 2mM glutamine is added for both GRA and MST. For Glycolytic Rate assay, 5mM HEPES is also added.

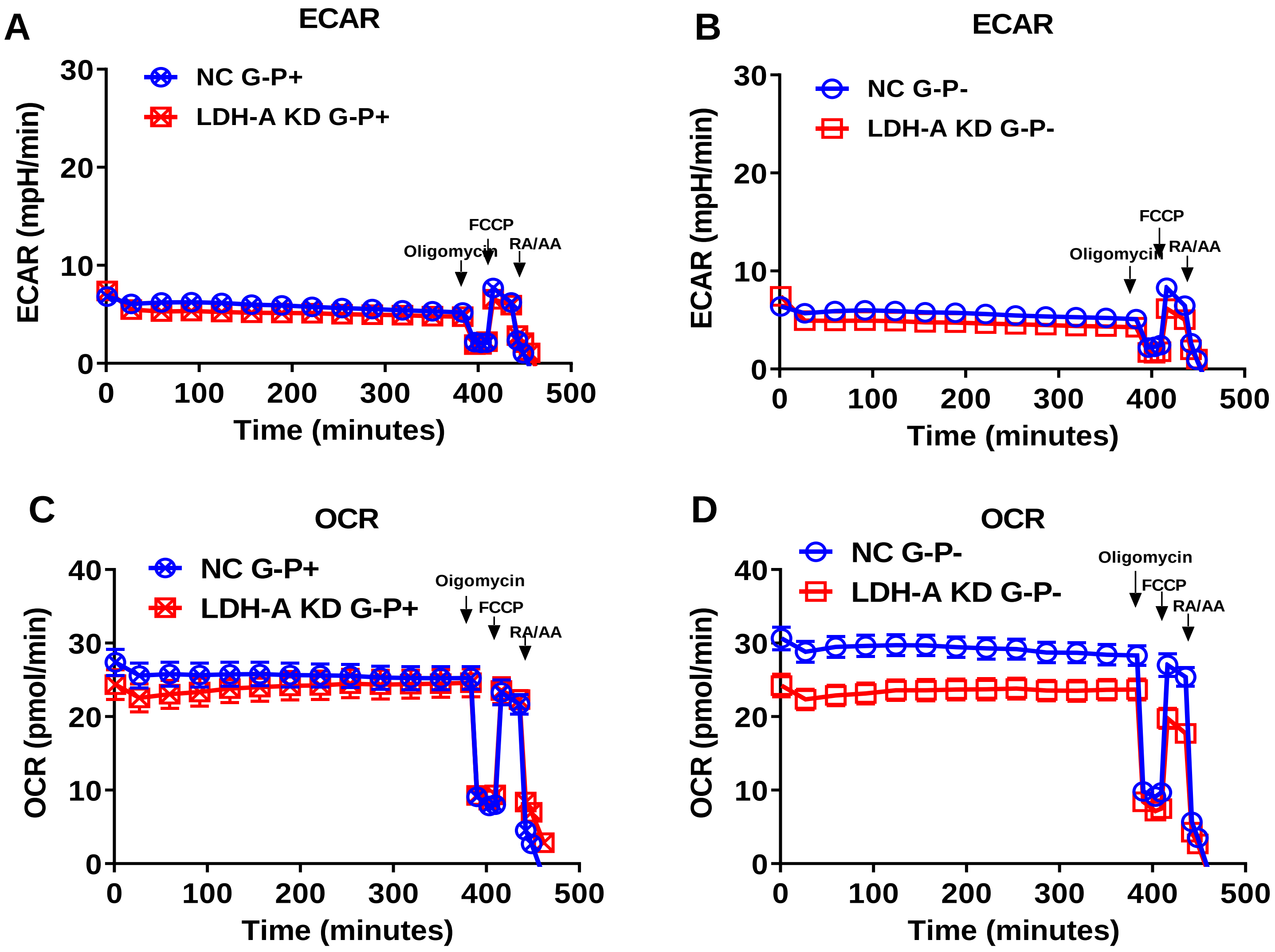

Figure S3. Metabolism of NC and LDH-A KD GL261 cells, in the presence and absence of Glucose and pyruvate. Real time profile analysis of the Extracellular Acidification Rate (ECAR) for NC and LDH-A KD GL261 cells, in the absence glucose (Glu-, 0 mM), and in the presence (1mM Na-pyruvate (Pyr+)) and absence (Pyr-, 0 mM) of Na-pyruvate (A-B). Real time profile analysis of the Oxidative Phosphorylation Rate (OCR) for NC and LDH-A KD GL261 cells, in the absence glucose (Glu-, 0 mM), and in the presence (1mM Na-pyruvate (Pyr+)) and absence (Pyr-, 0 mM) of Na-pyruvate (C-D). Values are Mean, ±SEM; n = 12.

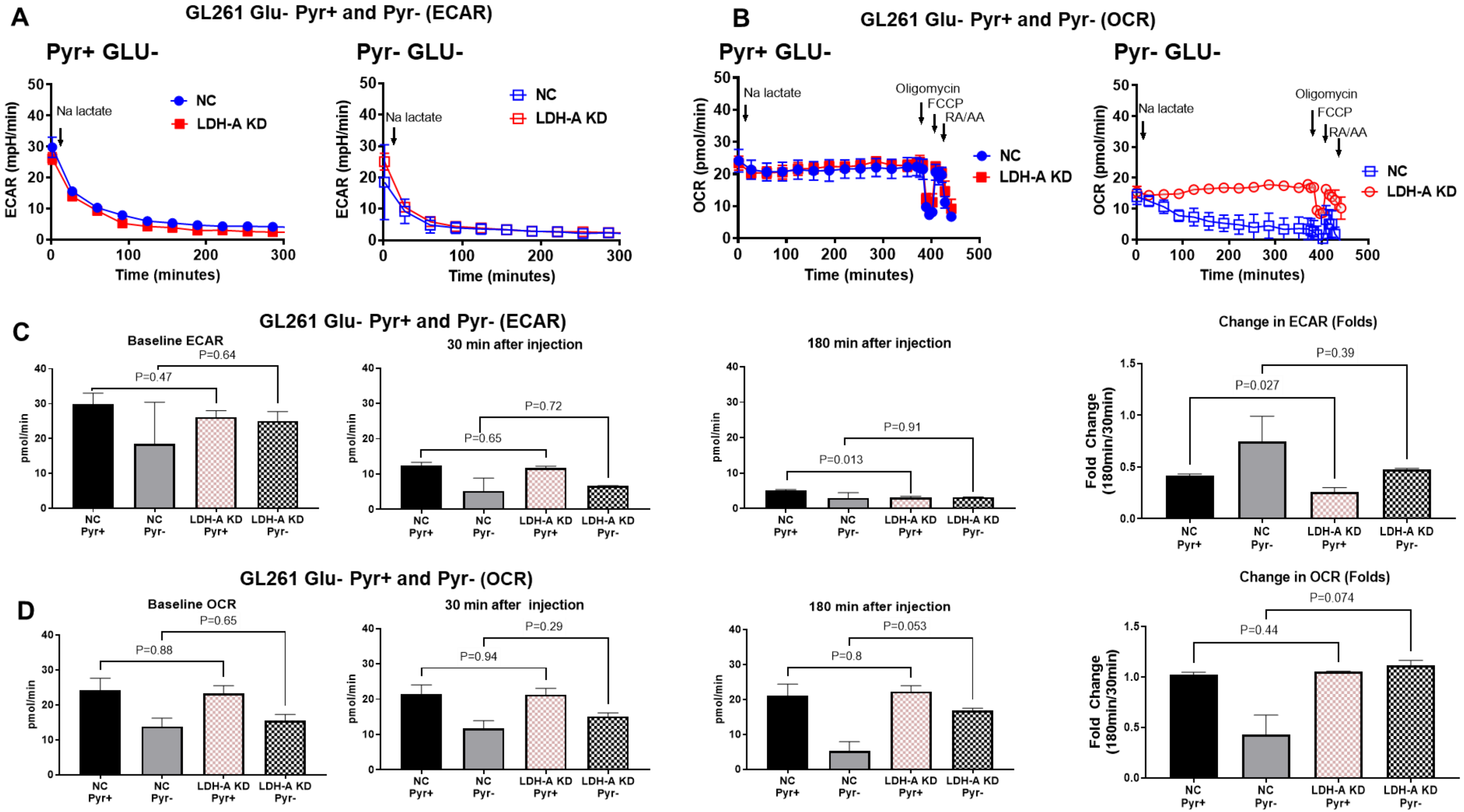

Figure S4. Effect of injected Na-Lactate on metabolism of NC and LDH-A KD GL261 cells, in the absence of glucose and in the presence or absence of pyruvate. Real time profile of the Extracellular Acidification Rate (ECAR) (**A**) and Oxidative Phosphorylation Rate (OCR) (**B**) in NC and LDH-A KD GL261 cells, on the addition of Na-Lactate to the experimental media, in presence (Pyr+) and absence (Pyr-) of 1mM Na-Pyruvate and in the absence of Glucose (glucose was absent in all experimental sets). Observed differences in Baseline ECAR (**C**) and OCR (**D**), as well as at 30 min and at 180 min following the addition of Na-Lactate to the media. Statistical significance of differences between NC and LDH-A KD cells, both in the presence and absence of 1mM Na-Pyruvate were assessed (summarized in Table S-2). Far right panels depict the changes in ECAR and OCR between 30 and 180 minutes (summarized in Table S-3). Values are mean, ±SEM; n = 2.

Table S3: Comparison of ECAR and OCR changes in GL261 cells following administration of 10 mM Na-Lactate to non-Glucose (0 mM) containing media, in the presence (Pyr+) and absence (Pyr-) of 1mM Pyruvate.

	GL261 cell line<br>In the absence of Glucose in media	P values for comparisons between groups	
		NC Pyr+ vs NC Pyr-	LDH-A KD Pyr+ vs LDH-A KD Pyr-
ECAR	Baseline ECAR	0.335	0.773
	30 min after injection	0.093	0.009
	180 min after injection	0.15	0.693
	Change in ECAR 30-180 min (Folds)	0.169	0.036
OCR	Baseline OCR	0.124	0.109
	30 min after injection	0.074	0.094
	180 min after injection	0.044	0.092
	Change in OCR 30-180 min (Folds)	0.028	0.316

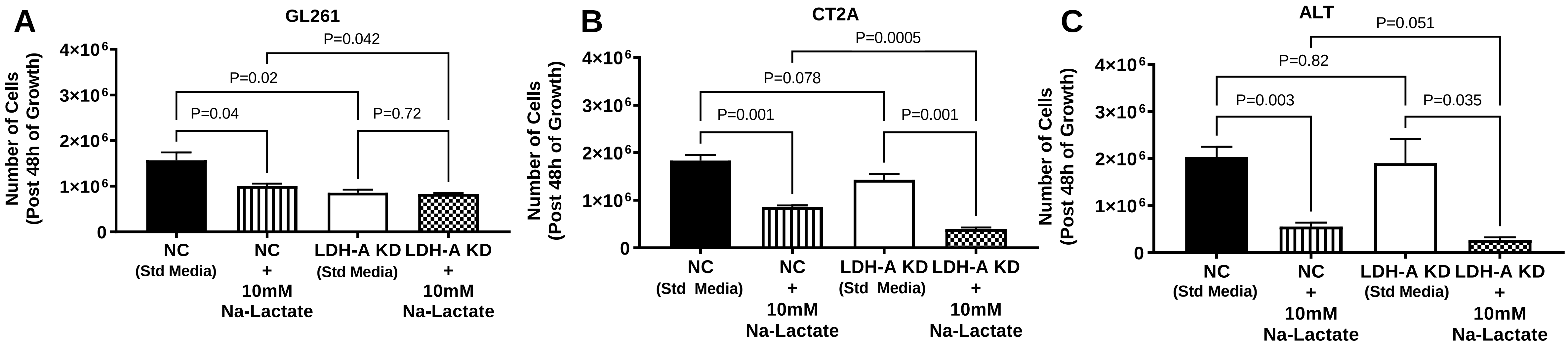

Figure S5. Effect of Na-Lactate on the proliferation of LDH-A KD and control NC glioma cell lines in the presence and absence of Glucose. Effect of adding 10 mM Na-Lactate to the culture media on the 48 hour proliferation of GL261 (**A**), CT2A (**B**) and ALTS1C1 (**C**) murine glioma cell lines (comparing LDH-A KD to the NC control; 6 cell lines). Values are mean, ± SEM; n = 3.

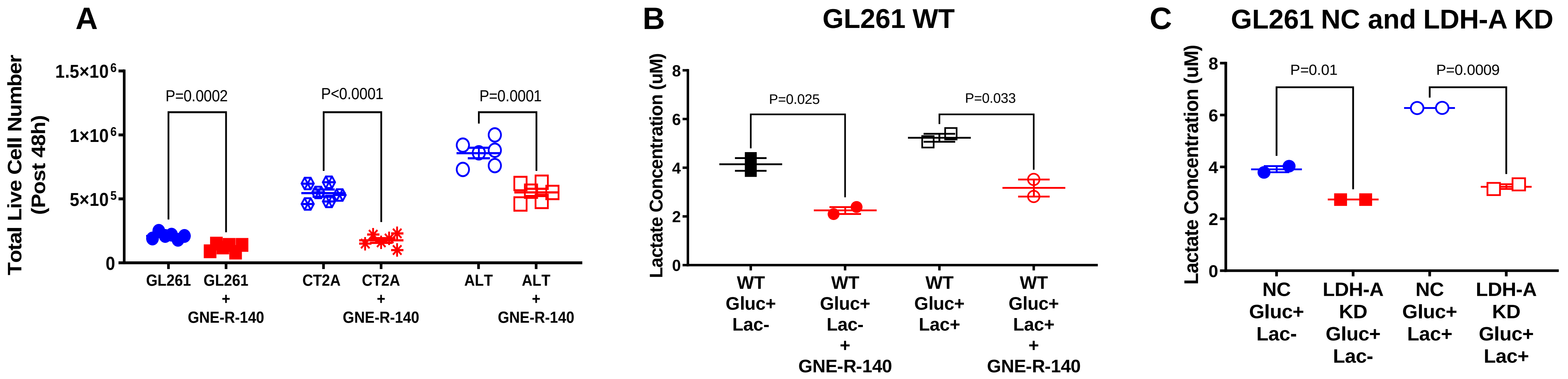

Figure S6. Effect of GNE-R-140 on cellular proliferation and intracellular lactate concentration. Effect of GNE-R-140 (10 µM) on proliferation of murine glioma cells GL261 WT, CT2A WT and ALTS1C1 WT over 48 h (**A**). *In vitro* intracellular lactate concentration changes both in the presence (Lac+) and absence (Lac-) of lactate, in 25 mM glucose-containing cell culture medium (Gluc+), with and without GNE-R-140 treatment of GL261 WT cells (**B**). Genetically modified GL261 cells with LDH-A KD show a similar effect, with lower intracellular lactate concentrations (**C**); mean ±SEM.

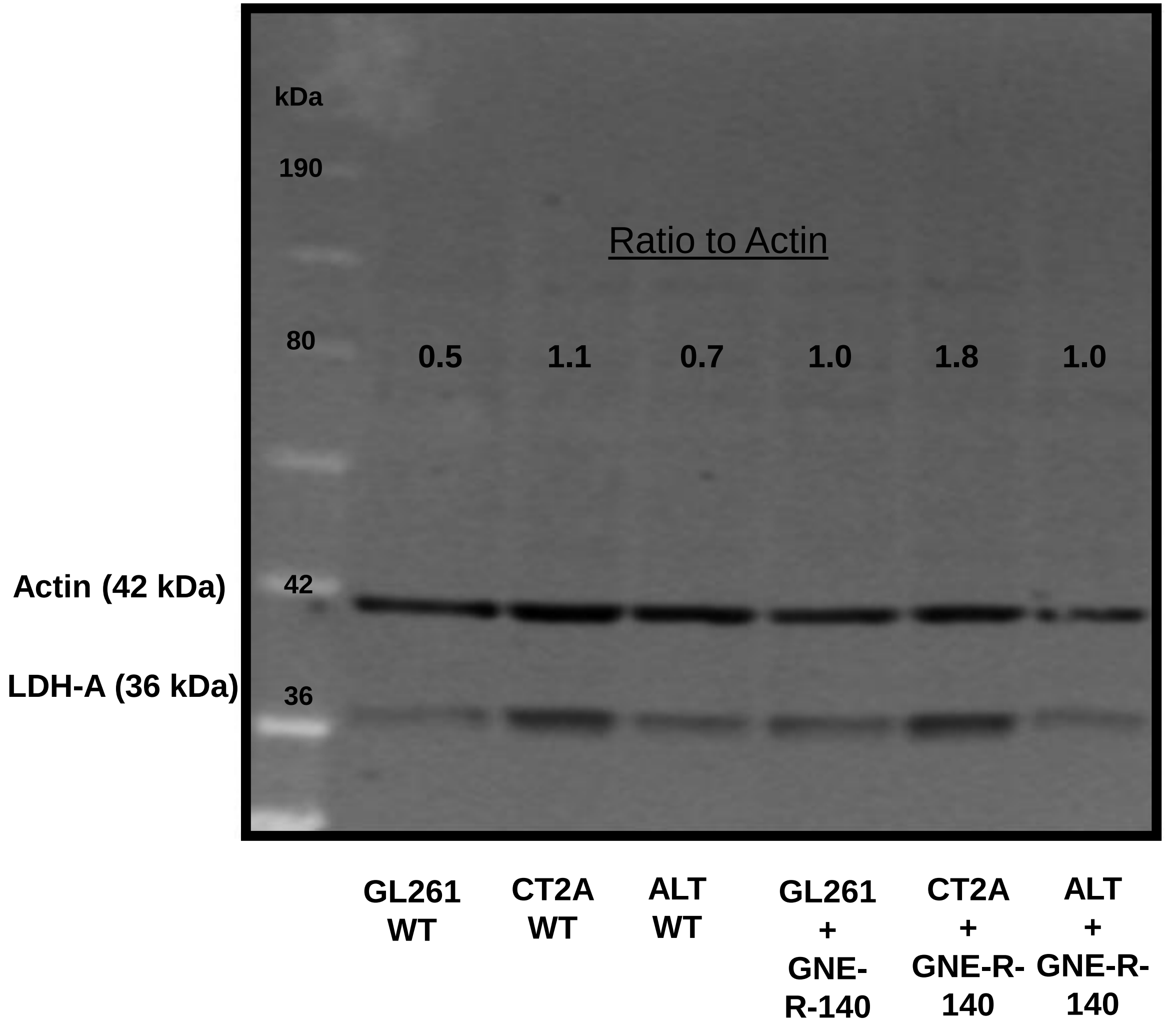

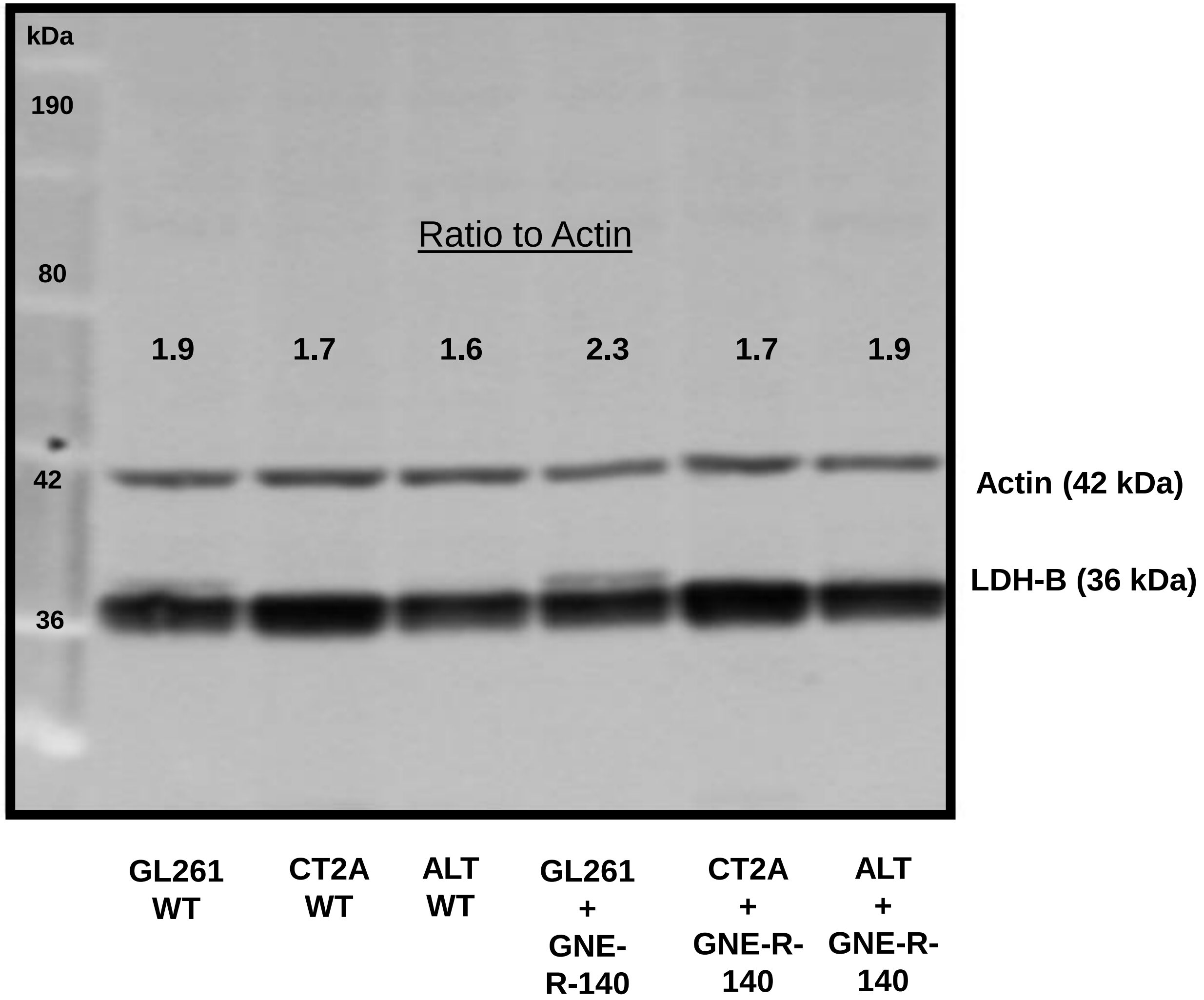

Figure S7. The native western blot for Panel A of Figure 6 is shown.
